# Type 2 Diabetes and Breast Cancer: The Interplay between Impaired Glucose Metabolism and Oxidant Stress

**DOI:** 10.1155/2015/183928

**Published:** 2015-06-11

**Authors:** Patrizia Ferroni, Silvia Riondino, Oreste Buonomo, Raffaele Palmirotta, Fiorella Guadagni, Mario Roselli

**Affiliations:** ^1^San Raffaele Rome University, IRCCS San Raffaele Pisana, Research Center, Via di Val Cannuta 247, 00166 Rome, Italy; ^2^Department of Systems Medicine, Medical Oncology, Tor Vergata Clinical Center, University of Rome Tor Vergata, Viale Oxford 81, 00133 Rome, Italy; ^3^Department of Surgery, Division of Surgical Oncology, Tor Vergata Clinical Center, University of Rome Tor Vergata, Viale Oxford 81, 00133 Rome, Italy

## Abstract

Metabolic disorders, especially type 2 diabetes and its associated complications, represent a growing public health problem. Epidemiological findings indicate a close relationship between diabetes and many types of cancer (including breast cancer risk), which regards not only the dysmetabolic condition, but also its underlying risk factors and therapeutic interventions. This review discusses the advances in understanding of the mechanisms linking metabolic disorders and breast cancer. Among the proposed mechanisms to explain such an association, a major role is played by the dysregulated glucose metabolism, which concurs with a chronic proinflammatory condition and an associated oxidative stress to promote tumour initiation and progression. As regards the altered glucose metabolism, hyperinsulinaemia, both endogenous due to insulin-resistance and drug-induced, appears to promote tumour cell growth through the involvement of innate immune activation, platelet activation, increased reactive oxygen species, exposure to protumorigenic and proangiogenic cytokines, and increased substrate availability to neoplastic cells. In this context, understanding the relationship between metabolic disorders and cancer is becoming imperative, and an accurate analysis of these associations could be used to identify biomarkers able to predict disease risk and/or prognosis and to help in the choice of proper evidence-based diagnostic and therapeutic protocols.

## 1. Introduction

Type 2 diabetes (T2D) constitutes a growing public health problem, with a global prevalence of 8.3% in 2013 (undiagnosed in approximately 30% of the cases), which is estimated to rise to more than 10% by 2035. Noteworthy, the largest increases will take place in developing countries, as T2D is epidemic in many low- and middle-income countries [1]. Although progression is not inevitable, impaired glucose tolerance (IGT, generally referred to as “prediabetes”) is a key condition in the evolution toward T2D. In 2013, global IGT prevalence has been estimated at 6.9%, a rate that is calculated to go up to 8% by 2035.

The relevance of these figures is evident if one considers that T2D is associated with a decrease of health-related quality of life and overall life expectancy and that T2D remains one of the leading causes of death, worldwide (especially due to cardiovascular complications). Looking at the 2013 estimates, in fact, T2D accounted for 8.4% of global all-cause mortality among individuals aged between 20 and 79 years, with an 11% increase over previous estimates for 2011 [1]. To further worsen this picture is the epidemiologic evidence of a close relationship between T2D and increased cancer risk [2–4], although an accurate assessment of cancer risk is complicated by the occurrence of several confounding factors such as disease duration, varying metabolic profiles, and the possible presence of shared cancer-promoting factors [5]. Accordingly, the American Association of Clinical Endocrinologists and the American College of Endocrinology highlighted in a recent joint consensus report the need for systemic studies to further investigate this relationship (AACE/ACE Consensus Statement) [6].

## 2. T2D and Breast Cancer Risk

T2D may affect multiple organs in different ways, and the female breast is not an exception. Of particular interest is a peculiar condition known as “diabetic mastopathy,” an infrequent proliferation of fibrous tissue in the breast parenchyma manifesting as unilateral or bilateral nodules [7]. Firstly described in 1984, diabetic mastopathy is a poorly characterized condition, often mistreated and not diagnosed as a diabetes complication. To date, there is no evidence of an association with breast cancer (BC), as the isolated case-reports of coexisting diabetic mastopathy and BC could merely represent two facets of concomitant diseases with high prevalence [7].

Beside this particular condition, increasing evidence suggest that diabetes contributes to BC risk. Up to 16% of BC patients, in fact, have T2D, which, in turn, has been associated with a 10–20% excessive risk of BC [8]. Moreover, several evidences indicate that T2D and impaired glucose tolerance may worsen BC prognosis [9, 10]. These figures were confirmed in a recent meta-analysis indicating an increased risk for BC of 23% in patients with T2D, and a 38% higher cancer-specific mortality risk in patients with T2D and BC. Accordingly, a positive association between T2D and BC incidence and mortality during a 10-year follow-up was also reported [11].

The possibility that an impaired glucose metabolism may influence BC incidence and outcomes clearly has major implications for primary and secondary prevention of BC. In this respect, it has been recently suggested that the variable prevalence of dysmetabolic conditions across Europe may contribute, at least in part, to the variations in BC survival across the continent, which cannot be completely explained by differences in stage at diagnosis or patient management [12, 13].

Based on molecular expression, BC can be classified into different subtype, either expressing hormone receptors, ER (oestrogen receptor), PR (progesterone receptor), and growth factor receptor HER2 (Human Epidermal Growth Factor Receptor) [14]. Clinical studies demonstrated that triple-negative BCs are associated with the poorest prognosis. In this regard, an independent association between HER2 and both hyperglycaemia and insulin resistance has been found [15], and circulating HER-2 concentrations seem to be significantly increased in T2D patients [16].

All these factors may act through independent and/or synergic mechanisms, either being responsible for a metabolic, hormonal, and inflammatory interplay (responsible for the more general association with the metabolic syndrome, as in the case of colorectal cancer [17]), or by acting in a site-specific manner, as in the case of female reproductive organs, and specifically in breast.

## 3. Impaired Glucose Regulation and Breast Cancer

**Figure 1:**
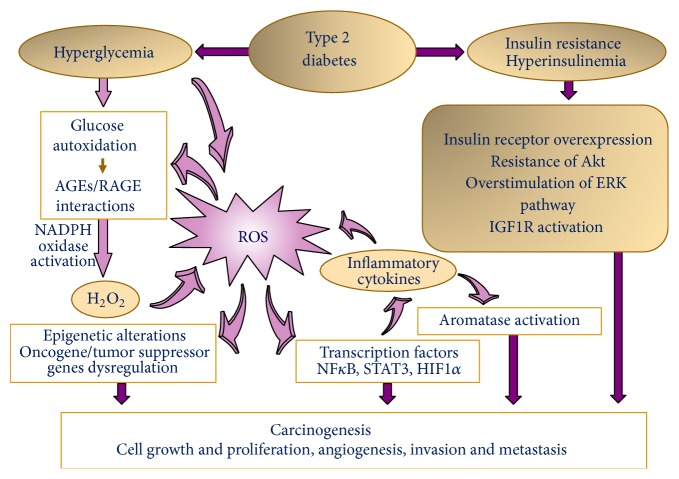
Pathways of oxidative stress associated with diabetes mellitus: mechanisms of carcinogenesis. Type 2 diabetes (T2D) causes both hyperglycaemia and hyperinsulinemia/IR. Hyperglycemia may induce reactive oxygen species (ROS) production directly via glucose metabolism and autooxidation and indirectly through the formation of advanced glycation end products (AGE) and their receptor (RAGE) binding. ROS, in turn, may exert their effects on DNA, through activation of signaling molecules (i.e., nuclear transcription factor-*κ*B—NF-*κ*B) and subsequent transcription of genes encoding cytokines and adhesive proteins. Hyperinsulinemia, insulin resistance (IR) and insulin-like growth factor-1 (IGF-1) activate signaling pathways, such as mitogen-activated protein kinase (MAPK) and AKT signaling pathways, that lead to carcinogenesis.

The possibility of a causal link between impaired glucose metabolism and cancer was initially raised by Marble in the first half of the 20th century [18]. Since then, several studies have investigated the possible etiological mechanisms underlying this association, demonstrating the central role of sustained hyperglycaemia, hyperinsulinemia, insulin resistance (IR), and hyperinsulinemia-related increase of insulin-like growth factor-1 (IGF-1) in cancer promotion and progression [19–22]. In turn, poor glycemic control, leads to a dysregulated metabolism, responsible for a long-term proinflammatory condition. In this scenario, an increasingly important role is played by chronic inflammation-induced oxidative stress that might concur with impaired glucose-associated conditions to promote tumour progression (Figure 1).

Hyperglycaemia is the hallmark for diabetes and results both from insufficient insulin production in pancreatic *β* cells, as in T1D, and from the increase of systemic insulin resistance, as in T2D [23]. Some authors reported a direct effect of glycaemia on cancer initiation, proliferation, migration, and invasiveness [24], and extensive research is presently available supporting a causative link between IGT/T2D and BC [25–29], which translates into a mild risk of breast carcinogenesis (a 1.2 risk ratio in the meta-analysis by Larsson et al. [4]) especially among postmenopausal women [30–32]. However, in patients with early-stage BC, T2D has been reported to be an independent predictor of lower BC-specific survival and, generally, of overall survival rates [33]. It is worthy to underline that the risk was independent of other conditions known to be associated with IR, such as obesity [32], thus supporting the involvement of site-specific mechanisms. These recognize, as a common denominator, the insulin axis. Insulin, in fact, is a potent regulator of human sexual steroid hormone synthesis that interferes with their signal transduction at cellular level [34, 35]. Indeed, in T2D patients, high levels of insulin reduces in the concentration of circulating sex hormone binding protein (SHBG) [36], thus leading to an increase in the levels of bioactive oestrogens which are responsible for the proliferation of both breast and endometrial cells, for the inhibition of apoptosis [37] and, possibly, for the enhancement of hormonal carcinogenesis [34, 38]. Furthermore, insulin and IGF-1 themselves are responsible for oestrogen production by enhanced expression of aromatase. Indeed, in obese T2D subjects, oestrone and estradiol are overproduced in the adipose tissue by the intense activity of aromatase [39], which has been found significantly expressed both in breast and in tumour tissues [40], and may fuel BC growth [38]. IGF-1, which participates in oestrogen receptor signalling via IGF-1 receptor/ER interaction, cooperates with oestrogens to regulate proliferation, apoptosis, and differentiation of mammary epithelial cells in a bidirectional way [35, 41]. Furthermore, the interaction between IGF-1 and 17*β*-estradiol results in the proliferation of breast carcinoma cells [42]. Therefore, it appears evident that insulin and oestrogen might have a mutual interrelationship capable of conferring high risk for endocrine-related cancers, especially in postmenopausal women [41].

The insulin/IGF axis is deeply involved in diabetes-associated increased risk and progression of cancer, to such an extent that it has been demonstrated that cancer cells overexpress both insulin and IGF-1 receptors [43, 44]. Physiologically, insulin exerts both metabolic and mitogenic effects, the former being mediated by phosphatidylinositol 3-kinase (PI3-K) pathway [45], whereas the latter is mainly achieved through mitogen-activated protein kinase (MAPK) pathway [34]. IGF-1 shares similar mechanisms of action, particularly in hyperinsulinemic conditions. In BC, they rather act as mitogens to enhance tumourigenesis via either pathway: insulin, via the insulin receptor substrate 1 (IRS-1) and IGF-1, by binding to its own receptor (IGF1R) [46] (Figure 1). It is worth noting that the activation of the PI3K pathways is required for insulin-induced upregulation of vascular endothelial growth factor (VEGF), which sustains neoangiogenesis and, thus, cancer progression [47]. Although the complex IGF-1/IGF1R shares high homology with insulin/insulin receptor [46], its activation results in a stronger favouring effect on BC cell proliferation and survival [48]. Due to this last evidence, in a condition of hyperinsulinemia, IGF1R can be activated both directly by the high circulating levels of insulin and, indirectly, through insulin-mediated upregulation of IGF-1 [48].

## 4. Chronic Inflammation and Oxidant Stress in T2D Converge in Breast Cancer

The consequences of hyperglycaemia on cancer cells behaviour can be either direct, as already reported, or indirect, through the increase in the levels of insulin/IGF-1 and inflammatory cytokines in circulation, such as interleukin-6 (IL-6) and tumour necrosis factor-alpha (TNF-*α*) [49], together with the most classical inflammatory markers such as C-reactive protein [3], but also through oxidative stress generation [50] and platelet activation [51]. Indeed, the two pathways of inflammation and oxidative stress seem to converge in the activation of nuclear factor *κ*B (NF*κ*B) [52], signal transducer and activator of transcription 3 (STAT3), and hypoxia-inducible factor 1*α* (HIF1*α*) [53]. These conditions are accompanied by an increase in free radicals, which can damage lipids and DNA both directly and indirectly and concur to promote oxidative stress and to amplify the inflammatory process [53] (Figure 1).

A condition of oxidative stress or of altered redox system is established following an unbalance between the production of reactive oxygen species (ROS) and/or reactive nitrogen species (RNS) and their removal by endogenous antioxidants [54]. ROS, like superoxide radical (O_2_^−^), the hydroxyl radical (HO^−^), and the nonradical hydrogen peroxide (H_2_O_2_), which normally participate to cell signalling, at high concentrations cause cell and tissue injury and damage. In T2D patients high levels of malondialdehyde (MDA), expression of the free radical-mediated lipid peroxidation, have been found associated with an increase in antioxidant enzymes possibly as a consequence of the adaptive response to prooxidant in diabetic state [55].

In this favorable milieu, ROS can initiate carcinogenesis by functioning as chemical effectors in the context of a redox unbalance [56], rendering cancer cells insensible to apoptosis, disrupting the cell anchorage sites and sustaining* de novo* angiogenesis [57]. It is widely acknowledged that ROS are key mediators of the metabolic coupling between the aerobic glycolysis in stromal cells (Warburg effect) and oxidative stress in cancer cells, which favours mitochondrial metabolism and tumourigenesis [58]. The changes in gene expression not resulting in DNA alteration, are commonly referred to as epigenetic and are regulated at several levels, among which are DNA methylation, histone modification and noncoding RNAs [53]. ROS have been implicated in both aberrant DNA hypermethylation and hypomethylation, and ROS-induced DNA methylation pattern alterations have been demonstrated both in malignant transformation and in cancer progression, thus representing important players in the epigenetic regulation in cancer cells (Figure 1) [59].

The association between T2D and cancer is further sustained by the elevated glycolytic rates and the formation of advanced glycation end products (AGEs) which, following interaction with their receptor (RAGE), leads to ROS generation, activation of NF-*κ*B and, finally, to cell damage (Figure 1). Conversely, the demonstration that blockade of RAGE-mediated signaling inhibits breast tumour growth and metastases, further evidences that RAGE expression is associated with BC [60].

As regards the inflammatory implications in BC, it has been demonstrated that tumour cells highly express also IL-8, IL-1*β* and monocyte chemoattractant protein-1 (MCP-1), whose levels are associated with poor recurrence-free survival in patients with HER2(−) tumours [61] and, in general, with poorer prognosis [50, 62]. Further evidence of a major role of cytokines in BC tumour progression comes from the demonstration that macrophages isolated from the tumour microenvironment of inflammatory BC patients secrete chemotactic cytokines that favour both dissemination and metastasis of carcinoma cells [63].

An important regulatory role is exerted by ROS in many cellular processes, among which the 3-phosphatase and tensin homolog (PTEN), which under normal conditions is a PI3-kinase inhibitor and, thus, acts on adhesion and motility [57]. It has been demonstrated that conditions of PTEN oxidation can result in BC promotion [57]. Genes encoding for proteins of the redox system are largely affected by oxidative stress, their polymorphism being responsible DNA damage, gene mutations, and, finally, carcinogenesis. The variations in genes from the stress oxidative pathway, such as polymorphism in exon 2 of the superoxide dismutase 2 (*SOD2*), catalase (*CAT*), and endothelial NO synthase (*eNOs*) genes, are, at various levels, involved in BC development [64].

In BC, a modulatory role has been established for the products of lipid peroxidation, hydroperoxides (HPs), 8-isoprostanes, and MDA in cancer initiation and progression [92–94]. In addition and in further support of this statement, it has been reported that advanced stages of the disease are characterized by a more pronounced oxidative status than earlier stages, with a marked reduction of the antioxidant enzyme catalase activity and an enhanced lipid peroxidation together with higher nitric oxide levels [94]. Furthermore, in patients with BC, lipid peroxidation profiling at diagnosis was significantly correlated with a 5-year recurrence, following tumour removal, possibly leading to relapse or metastatic disease [94].

## 5. Effects of Antidiabetic Treatment on Breast Cancer Initiation and Progression

Of course, talking about the association between T2D and BC we must take into consideration the causative association between T2D and BC deriving from clinical trials reporting the effects of antidiabetic medications. Insulin, insulin analogues and secretagogues (all acting through an increase of the circulating levels of insulin) have been associated with increased risk of cancer [95]. Insulin treatment, in particular, was investigated in a recent meta-analysis of 10 cohort studies, demonstrating a combined risk ratio (RR) of 1.28 (95% CI: 1.03, 1.59) and individual RRs ranging from 1.19 to 3.87 [95]. However, when BC was investigated separately, inconsistent results were observed [66, 74]. Other glucose-lowering drugs, such as sulfonylureas and glinides acting through sustained insulin production, have been investigated, but data are conflicting and deeper investigation is required to substantiate their possible association with increased cancer risks [95].

Metformin, in turn, was constantly associated with a reduced risk of cancer due not only to indirect mechanisms related to inhibition of hepatic gluconeogenesis and reduced insulin signaling via inhibition of phosphoinositide 3-kinase (PI3K) cellular response, but also to direct mechanisms operating through the tumour suppressor protein, LKB1, mediated activation of the AMP-activated protein kinase (AMPK) pathway and consequent suppression of energy stress response ultimately affecting cancer cell survival [96]. Indeed, in neoplastic cells, the increased AMPK activity leads to downstream inhibition of PI3K/Akt/mammalian target of rapamycin (mTOR) and MEK/ERK1/2mTOR signaling, protein synthesis, and proliferation [19]. Metformin can also directly inhibit tumour cell growth by modulating cyclin D1-medicated cell cycle and the expression of tumour suppressor p53 in different tumour cells including breast carcinoma cells [97, 98]. Another mechanism reported for cell apoptosis and death mediated by metformin, is by increasing oxidative stress, following AMPK and forkhead transcription factor 3 (FOXO3) protein activation [98], and by increasing activities of antioxidant molecules, such as Cu-Zn, SOD, catalase, and GSH in the erythrocytes [53]. As a consequence, this would render the erythrocytes less prone to oxidative stress [53].

The benefits of the reduction in insulin levels in T2D or hyperinsulinaemic cancer patients, whose tumour growth is under the influence of insulin, may account for the suggestion to use metformin in this subset of patients. The capability of metformin to increase apoptosis of BC cells* in vitro* has been documented in experimental models employing wild-type, tamoxifen-resistant, and oestrogen-deprived MCF-7 cells [99]. Interestingly, the reduction of neoplastic growth was more pronounced when metformin and tamoxifen were used in combination [99]. Thus, metformin-induced pleiotropic effects might be effective in enhancing the activities of the currently available hormonal therapies.

Overall, several meta-analysis of metformin clinical trials demonstrated a substantial reduction of cancer risk (approximately 40%) compared with no use (Table 1) [73, 75–78]. However, when specific cancer sites were analyzed separately, reduced risk was confirmed in colorectal and pancreatic cancers, whereas discordant results were reported for metformin use and BC risk [73, 75–78]. Looking into the single studies reported in the meta-analyses, pioneering retrospective investigations showed a nonsignificant association between metformin use and BC risk [65, 66], which was confirmed in subsequent case-control studies (Table 1) [67–72], except when analyses were performed to estimate the impact of different duration metformin use [71]. Beside an increased BC risk, several evidences suggest a positive impact of metformin treatment on breast cancer survival outcomes, especially in particular subset of patients (Table 2) [82–91], at a point that this drug is currently being investigated for its effects on invasive disease-free survival and other outcomes in a phase III randomized trial in early BC (Clinical Trials.gov identifier: NCT01101438, estimated study completion date: December 2017).

Finally, thiazolidinediones (TZDs, namely, pioglitazone and rosiglitazone), introduced as oral antidiabetic agents to directly target insulin resistance, have been recently investigated for possible association with cancer risk, mainly because of the public health alert issued by the EMA concerning an increased risk of bladder cancer among diabetic patients treated with pioglitazone [100]. Despite this association, a meta-analysis performed by Colmers et al. demonstrated that TZDs use was related to a modest but significantly reduced risk of BC (RR: 0.89, 95% CI 0.81–0.98) [79]. These results were confirmed in a recent six-year population-based cohort study showing a dose dependent decrease in specific cancer risks in diabetic patients using TZDs [80]. Furthermore, the results of a retrospective analysis of the electronic health record-based Cleveland Clinic Diabetes Registry cross-indexed with the histology-based tumour registry over an 8-year period, clearly demonstrated that TZDs use in women was associated with a 32% decreased cancer risk compared with sulphonylurea [81].

## 6. Conclusions and Perspectives

Looking back on what has been discussed, it appears clear that T2D should be looked at as a hidden enemy and that understanding the association between diabetes, oxidative stress, and site-specific cancers is becoming imperative and efforts should be employed to improve screening measures and to develop risk assessment tools. Oxidative stress, indeed, besides being responsible for the damage-induced development of cancer, represents a key steps involved in the mutagenesis that leads to carcinogenesis and might be responsible for the redox adaptation of cancer cells that become resistant to anticancer agents. This appears even more mandatory in an aging world in which sociodemographic, epidemiological, and technological factors are responsible for an increase in life expectancy and a higher health care demand for the National Health Systems (NHS). Older people, in fact, are more vulnerable to noncommunicable diseases (typically T2D and cancer) resulting in a considerable impact on the NHS, as more resources are needed to warrant appropriate standards of medical care and improve quality of life. In this context, the knowledge that the rate of biological aging is at least partially modulated by genes interacting with stressor exposures [101] is of utmost importance and, after more than 50 years, the mitochondrial free radical theory of aging is still endorsed [102]. Despite the conflicting outcomes of nontargeted antioxidants in clinical trials, there is growing evidence that helping with lifestyle interventions (including physical activity, dietary modifications, and appropriate therapeutic strategies, counting also supportive antioxidant supplementation ones) might have a clinically relevant role in reducing BC risk or progression in postmenopausal women with T2D. Finally, it becomes imperative for the clinicians to consider all comorbidities when dealing with diabetic patients with cancer, in which outcomes of both disease and chemotherapy may result in a poorer prognosis.

## Figures and Tables

**Table 1:** Metformin and thiazolidinediones use and breast cancer risk in type 2 diabetes.

Reference	Study design	Number of cases/controls	Mean age (years)	Treatment comparison	Risk estimates (95% CI)
Metformin use: clinical studies					
Libby et al., 2009 [65]	Population-based, historical cohort study	771/8170	66	Non-metformin users	0.6 (0.32–1.10)
Currie et al., 2009 [66]	General practices, retrospective cohort study	373/7897	64	Sulfonylureas monotherapy Insulin-based treatments	1.02 (0.71–1.45) 0.93 (0.69–1.27)
Bodmer et al., 2010 [67]	Nested case-control study	17/120	68	Non-metformin users	0.44 (0.24–0.82)
Bosco et al., 2011 [68]	Nested case-control study	393/3930	>50 years	Non-metformin users	0.81 (0.63–0.96)
Ruiter et al., 2012 [69]	Case-control study	207/217	NA	Sulfonylureas	0.95 (0.91–0.98)
Chlebowski et al., 2012 [70]	Observational cohort	104/129	64	Other antidiabetic drugs	0.75 (0.57–0.99)
van Staa et al., 2012 [71]	Observational cohort/inception cohorts	160/86 268/86 184/86	63	Metformin treatment <6 months	0.73 (0.56–0.96)^a^ 0.91 (0.70–1.17)^b^ 0.82 (0.61–1.10)^c^
García-Esquinas et al., 2015 [72]	Population-based multicase-control study	24/43	NA	Duration of metformin use	0.89 (0.81–0.99) for ER+/PR+ Her2−

Metformin use: meta-analyses					
DeCensi et al., 2010 [73]	Meta-analysis of various cancers, including [65, 74, 75]		NA	NA	0.70 (0.28–1.77)
Soranna et al., 2012 [76]	Meta-analysis of various cancers, including [65, 67, 74, 75]		NA	NA	0.87 (0.69–1.10)
Franciosi et al., 2013 [77]	Meta-analysis of various cancers, including [65, 67–70, 74, 75]		NA	NA	0.71 (0.58–0.88)
Col et al., 2012 [78]	Meta-analysis of breast cancer studies, including [65, 67–70, 74, 75]		NA	NA	0.83 (0.71–0.97)
Zhang et al., 2013 [75]	Meta-analysis of various cancers, including [65, 67–69, 74, 75]		NA	NA	0.94 (0.91–0.97)

Thiazolidinedione (TZD) use					
Bodmer et al., 2010 [67]	Nested case-control study	12/30	68	TZD long-term users	1.76 (0.84–3.68)
Colmers et al., 2012 [79]	Meta-analysis of various cancers		NA	NA	0.89 (0.81–0.98)
van Staa et al., 2012 [71]	Observational cohort/inception cohorts	160/86 268/86 184/86	63	TZD treatment <6 months	1.04 (0.60–1.80)^a^ 0.99 (0.56–1.75)^b^ 1.09 (0.53–2.22)^c^
Lin et al., 2014 [80]	Population-based retrospective cohort study	NA	56	Other antidiabetic drugs No antidiabetic drugs	0.22 (0.09–0.55) 0.19 (0.07–0.54)
Sun et al., 2014 [81]	Retrospective cohort study	NA	66	Sulphonylurea	0.68 (0.48–0.97)^d^

^a^6–24 months since start of metformin/TZD.

^b^25–60 months since start of metformin/TZD.

^c^ >60 months since start of metformin/TZD.

^d^All women cancer types.

**Table 2:** Metformin use and survival outcomes of breast cancer patients.

Reference	Study design	Study population^a^	BC type	Mean age (years)	Metformin versus non-metformin	Study findings
Currie et al., 2012 [82]	Retrospective	1182	All	NA	NA	OS: HR 0.96 (95% CI: 0.67–1.37)

Bayraktar et al., 2012 [83]	Retrospective	130	Triple negative	52	63 versus 67	RFS: HR: 1.37 (95% CI: 0.78–2.40) OS: HR: 1.22 (95% CI: 0.66–2.28)

He et al., 2012 [84]	Retrospective	154	HER2+	55	88 versus 66	OS: HR 0.52 (95% CI: 0.28–0.97)

Peeters et al., 2013 [85]	Retrospective	1058	All	NA	508 versus 550	OS: HR 0.74 (95% CI: 0.58–0.96)

Lega et al., 2013 [86]	Population-based	2361	All	77	1094 versus 1267	OS: HR 0.97 (95% CI: 0.92–1.02)

Hou et al., 2013 [87]	Retrospective	1013	All	NA	419 versus 594	NA^b^

Oppong et al., 2014 [88]	Retrospective	145	All	61	76 versus 65	RFS: HR 0.86 (95% CI: 0.38–1.90)

Xiao et al., 2014 [89]	Retrospective	680	Luminal	NA	275 versus 405^c^	OS luminal A: HR 3.58 (95% CI: 1.51–8.51) OS luminal B (Ki67): HR 3.23 (95% CI: 1.84–5.68) OS luminal B (Her2+): HR 2.034 (95% CI: 1.02–4.06)

Kim et al., 2015 [90]	Retrospective	386	ER/PR status Her2 status	55/59	202 versus 184	CSS ER+/PR+ Her2+: HR 6.51 (95% CI: 2.06–20.6) DFS ER+/PR+ Her2+: HR 5.37 (95% CI: 1.88–15.3)

Vissers et al., 2015 [91]	Retrospective	1057	All	71	688 versus 369	OS: HR 0.47 (95% CI: 0.26–0.82)

BC: breast cancer; NA: not available; OS: overall survival; HR: hazard ratio; RFS: relapse-free survival; ER: estrogens receptors; PR: progesterone receptors; CSS: cancer specific survival; DFS: disease-free survival.

WHI: Women's Health Initiative, comprising four clinical trials and an observational study; ^a^Including only breast cancer patients with diabetes; ^b^HR 0.76 (95% CI: 0.6–0.99) for OS of metformin-treated patients compared to nondiabetic patients.

^c^Non-metformin versus metformin group.
